# Draft Genome Sequences and Antimicrobial Resistance Genes of Five Staphylococcus aureus Strains Isolated from Bovine Milk

**DOI:** 10.1128/mra.00756-22

**Published:** 2022-10-03

**Authors:** Shoaib Ashraf, Sohail Naushad, Wei Si, Muhammad Bilal, Muhammad Ijaz, Hongsheng Huang, Xin Zhao

**Affiliations:** a Department of Animal Science, McGill University, Sainte-Anne-de-Bellevue, Quebec, Canada; b Ottawa Laboratory-Fallowfield, Canadian Food Inspection Agency, Ottawa, Ontario, Canada; c Institute of Animal Sciences of the Chinese Academy of Agricultural Sciences, Beijing, China; d Department of Veterinary Medicine, University of Veterinary and Animal Sciences, Lahore, Pakistan; University of Rochester School of Medicine and Dentistry

## Abstract

Staphylococcus aureus is one of the most important bacterial pathogens causing bovine mastitis, which leads to huge economic losses worldwide. Here, we report draft genome sequences and antimicrobial resistance gene profiles of five Staphylococcus aureus strains that were isolated from bovine milk in Pakistan.

## ANNOUNCEMENT

Staphylococcus aureus continues to be recognized as one of the major and most prevalent agents of dairy cow intramammary infections worldwide (1, 2). We report draft genome sequences of five S. aureus strains, three of which were isolated from buffalo milk and two of which were isolated from milk samples from cattle in Pakistan. Milk samples were collected from buffalo and cattle in 2016. For bacterial isolation and identification, milk samples were initially cultured on blood agar (Oxoid). Then, colonies were streaked on mannitol salt agar (Oxoid), and yellow colonies were picked, after which S. aureus was confirmed by Gram staining and biochemical tests (coagulase, catalase, and mannitol fermentation tests). The strains were propagated in tryptic soy broth (Oxoid). All cultures were incubated at 30°C for 24 h.

Genomic DNA (gDNA) was extracted from overnight cultures from a single colony grown overnight on tryptic soy agar plates, using the DNeasy blood and tissue kit (Qiagen) according to the protocol for Gram-positive bacteria. The quantity and quality of gDNA were checked with a Qubit fluorometer and a NanoDrop spectrophotometer (Thermo Fisher Scientific), respectively. DNA was diluted to a final concentration of 0.2 ng/μL for paired-end Illumina library preparation using the Nextera XT library preparation kit (Illumina). All sequencing steps, including cluster generation, paired-end sequencing (2 × 300 bp), and primary data analysis for quality control, were performed on the Illumina MiSeq platform. Poorly sequenced regions and Illumina adapters from the sequenced reads were checked and trimmed with fastp v0.23.2, and *de novo* assembly of filtered reads into contigs was performed with SKESA v2.4.0 (3). The depth of sequencing coverage was computed by aligning filtered reads back to assembled contigs using BWA v0.7.17 (4) and SAMtools (5), and the results were analyzed using Qualimap v2.2.1 (6). Gene prediction and annotation were performed using the NCBI Prokaryotic Genome Annotation Pipeline (PGAP) v6.0 (7). Antimicrobial resistance (AMR) genes were identified using RGI v5.2.0 (8), and only perfect (100% identity match with curated reference sequences and mutations in the Comprehensive Antibiotic Resistance Database [CARD]) and strict (≥95% identity) hits for AMR genes were retained. Default parameters were used for all software unless otherwise specified.

Each strain contains 14 to 18 contigs, with an average assembled size of 2,744,080 bp; the strains had 119× coverage on average and 55 tRNAs, and the assembled contigs had an average *N*_50_ value of 433,967 bp (Table 1). The annotated genomes had on average 2,611 coding sequences (CDSs) and a GC content of 32.72%, similar to the average for S. aureus strains in the NCBI database (accessed 22 July 2022). The total length of the assembled genome, the number of CDSs, the GC content, and the number of tRNAs for each strain are provided in Table 1. Multiple AMR genes were detected in each assembled genome. Fifteen AMR genes [*norA*, *mgrA*, *arlR*, *tet*(38), *mepR*, *mepA*, *lmrS*, *arlS*, *dfrG*, *tet*(K), *ant(4′*)-*Ib*, *lnuA*, PC1 *β-lactamase* (*bla_Z_*), *mphC*, and *msrA*] were detected, and their distribution in each genome is presented in Table 1.

**TABLE 1:** Genomic characteristics and AMR genes of the five S. aureus strains isolated from bovine milk

S. aureus strain	Source	Total no. of reads	Assembled genome size (bp)	Assembly *N*_50_ (bp)	Genome coverage (×)	Total no. of contigs	No. of CDSs (with protein)	GC content (%)	No. of tRNAs	GenBank assembly accession no.	SRA accession no.	AMR genes
P4	Buffalo milk	812,179	2,734,808	442,789	138	14	2,593	32.74	55	GCA_024178415.1	SRR20055008	*norA*, *mgrA*, *arlR*, *tet*(38), *mepR*, *mepA*, *lmrS*, *arlS*
P5	Buffalo milk	607,206	2,734,028	324,514	103	18	2,601	32.74	55	GCA_024178345.1	SRR20055006	*norA*, *mgrA*, *arlR*, *tet*(38), *mepR*, *mepA*, *lmrS*, *arlS*
P7	Buffalo milk	637,635	2,736,349	443,363	107	15	2,598	32.73	55	GCA_024178475.1	SRR20055004	*norA*, *mgrA*, *arlR*, *tet*(38), *mepR*, *mepA*, *lmrS*, *arlS*
P18	Cattle milk	713,010	2,737,569	386,823	121	16	2,598	32.73	55	GCA_024178405.1	SRR20055002	*norA*, *mgrA*, *arlR*, *tet*(38), *mepR*, *mepA*, *lmrS*, *arlS*
P23	Cattle milk	763,090	2,777,647	622,344	128	15	2,666	32.66	55	GCA_024178305.1	SRR20055000	*lmrS*, *mepR*, *dfrG*, *arlR*, *arlS*, *mgrA*, *tet*(K), *mepA*, *tet*(38), *norA*, *ant(4')-Ib*, *lnuA*, PC1 β-*lactamase* (*bla_Z_*), *mphC*, *msrA*

### Data availability.

The assembled genome sequences of the five isolates and their MiSeq-sequenced reads in fastq format have been deposited in GenBank under BioProject accession number PRJNA854047. Accession numbers are provided in Table 1.

## References

[1] Naushad S, Nobrega DB, Naqvi SA, Barkema HW, De Buck J. 2020. Genomic analysis of bovine *Staphylococcus aureus* isolates from milk to elucidate diversity and determine the distributions of antimicrobial and virulence genes and their association with mastitis. mSystems 5:e00063-20. doi:10.1128/mSystems.00063-20. 32636332 PMC7343304

[2] Campos B, Pickering AC, Rocha LS, Aguilar AP, Fabres-Klein MH, de Oliveira Mendes TA, Fitzgerald JR, de Oliveira Barros Ribon A. 2022. Diversity and pathogenesis of *Staphylococcus aureus* from bovine mastitis: current understanding and future perspectives. BMC Vet Res 18:115. doi:10.1186/s12917-022-03197-5. 35331225 PMC8944054

[3] Souvorov A, Agarwala R, Lipman DJ. 2018. SKESA: strategic k-mer extension for scrupulous assemblies. Genome Biol 19:153. doi:10.1186/s13059-018-1540-z. 30286803 PMC6172800

[4] Li H, Durbin R. 2010. Fast and accurate long-read alignment with Burrows-Wheeler transform. Bioinformatics 26:589–595. doi:10.1093/bioinformatics/btp698. 20080505 PMC2828108

[5] Li H, Handsaker B, Wysoker A, Fennell T, Ruan J, Homer N, Marth G, Abecasis G, Durbin R, 1000 Genome Project Data Processing Subgroup. 2009. The Sequence Alignment/Map format and SAMtools. Bioinformatics 25:2078–2079. doi:10.1093/bioinformatics/btp352. 19505943 PMC2723002

[6] Okonechnikov K, Conesa A, García-Alcalde F. 2016. Qualimap 2: advanced multi-sample quality control for high-throughput sequencing data. Bioinformatics 32:292–294. doi:10.1093/bioinformatics/btv566. 26428292 PMC4708105

[7] Tatusova T, DiCuccio M, Badretdin A, Chetvernin V, Nawrocki EP, Zaslavsky L, Lomsadze A, Pruitt KD, Borodovsky M, Ostell J. 2016. NCBI Prokaryotic Genome Annotation Pipeline. Nucleic Acids Res 44:6614–6624. doi:10.1093/nar/gkw569. 27342282 PMC5001611

[8] Alcock BP, Raphenya AR, Lau TTY, Tsang KK, Bouchard M, Edalatmand A, Huynh W, Nguyen AV, Cheng AA, Liu S, Min SY, Miroshnichenko A, Tran HK, Werfalli RE, Nasir JA, Oloni M, Speicher DJ, Florescu A, Singh B, Faltyn M, Hernandez-Koutoucheva A, Sharma AN, Bordeleau E, Pawlowski AC, Zubyk HL, Dooley D, Griffiths E, Maguire F, Winsor GL, Beiko RG, Brinkman FSL, Hsiao WWL, Domselaar GV, McArthur AG. 2020. CARD 2020: antibiotic resistome surveillance with the Comprehensive Antibiotic Resistance Database. Nucleic Acids Res 48:D517–D525. doi:10.1093/nar/gkz935. 31665441 PMC7145624

